# 4-Pyridone-3-carboxamide-1-β-D-ribonucleoside Triphosphate (4PyTP), a Novel NAD^+^ Metabolite Accumulating in Erythrocytes of Uremic Children: A Biomarker for a Toxic NAD^+^ Analogue in Other Tissues?

**DOI:** 10.3390/toxins3060520

**Published:** 2011-06-07

**Authors:** Elena Synesiou, Lynnette D. Fairbanks, H. Anne Simmonds, Ewa M. Slominska, Ryszard T. Smolenski, Elizabeth A. Carrey

**Affiliations:** 1 University College London Institute of Child Health, 30 Guilford Street, London WC1N 1EH, UK; 2 Purine Research Laboratory, Chemical Pathology Department, St Thomas’ Hospital, London SE1 7EH, UK; Email: Lynette.Fairbanks@gsts.com (L.D.F.); 3 Department of Biochemistry, Medical University of Gdansk, Debinki 1, 80-211 Gdansk, Poland; Email: eslom@gumed.edu.pl (E.M.S.); rt.smolenski@gmail.com (R.T.S.)

**Keywords:** uremia, erythrocytes, pyridone, NAD^+^, 4-pyridone 3/5-carboxamide ribonucleoside triphosphate (4PyTP), nicotinamide riboside (NR), IMP dehydrogenase, HPLC

## Abstract

We have identified a novel nucleotide, 4-pyridone 3/5-carboxamide ribonucleoside triphosphate (4PyTP), which accumulates in human erythrocytes during renal failure. Using plasma and erythrocyte extracts obtained from children with chronic renal failure we show that the concentration of 4PyTP is increased, as well as other soluble NAD^+^ metabolites (nicotinamide, *N*^1^-methylnicotinamide and 4Py-riboside) and the major nicotinamide metabolite *N*^1^-methyl-2-pyridone-5-carboxamide (2PY), with increasing degrees of renal failure. We noted that 2PY concentration was highest in the plasma of haemodialysis patients, while 4PyTP was highest in erythrocytes of children undergoing peritoneal dialysis: its concentration correlated closely with 4Py-riboside, an authentic precursor of 4PyTP, in the plasma. In the dialysis patients, GTP concentration was elevated: similar accumulation was noted previously, as a paradoxical effect in erythrocytes during treatment with immunosuppressants such as ribavirin and mycophenolate mofetil, which deplete GTP through inhibition of IMP dehydrogenase in nucleated cells such as lymphocytes. We predict that 4Py-riboside and 4Py-nucleotides bind to this enzyme and alter its activity. The enzymes that regenerate NAD^+^ from nicotinamide riboside also convert the drugs tiazofurin and benzamide riboside into NAD^+^ analogues that inhibit IMP dehydrogenase more effectively than the related ribosides: we therefore propose that the accumulation of 4PyTP in erythrocytes during renal failure is a marker for the accumulation of a related toxic NAD^+^ analogue that inhibits IMP dehydrogenase in other cells.

## Abbreviations

2PY and 4PY: *N*^1^-methyl-2-pyridone-5-carboxamide and *N*^1^-methyl-4-pyridone-3/5-carboxamide
4KNTP: 4-ketonicotinamide riboside triphosphate *alias* 4PyTP
4Py-riboside: the *N*^1^-ribonucleoside of 4-pyridone-3/5-carboxamide
4PyTP, 4PyDP, 4PyMN: the triphosphate, diphosphate and mononucleotide of 4-pyridone-3/5-carboxamide-1β-D-ribonucleoside
GFR: glomerular filtration rate for creatinine
HD: (mechanical) haemodialysis
IMPDH: inosine monophosphate dehydrogenase, (EC 1.1.1.205, IMP:NAD oxidoreductase)
N-Me-nic: *N*^1^-methylnicotinamide
NAD^+^: nicotinamide adenine dinucleotide
NMN: nicotinamide mononucleotide
NR: nicotinamide riboside
PCNR: 4-pyridone carboxamide ribonucleoside (*alias* 4Py-riboside)
PD: patients undergoing continuous ambulatory peritoneal dialysis
*r*: Pearson correlation coefficient

## 1. Introduction

The nicotinamide catabolite *N*^1^-methyl-2-pyridone-5-carboxamide (2PY), derived from the breakdown of NAD^+^ during natural turnover and in response to DNA damage [1], is found in the urine of normal people, and also at high concentrations in the plasma of patients with renal failure [2]. We reported previously that a novel pyridone nucleotide, detectable in erythrocytes of healthy people, also accumulates to high concentrations in end-stage renal failure, and may reach concentrations of 0.5 mM in the erythrocytes of adult patients receiving renal replacement therapy [3]. The nucleotide elutes after ATP in our anion-exchange HPLC profiles, and has a characteristic UV absorption spectrum similar but not identical to that of 2PY [4]. The natural compound (tentatively named 2PyTP [3]) has now been identified unequivocally as the *N*^1^-riboside triphosphate of 4-pyridone 3/5-carboxamide (4PyTP) [5], earlier also named by us as 4-ketonicotinamide riboside triphosphate or 4KNTP [6].

In this study we measured the concentrations of 2PY (principally in plasma) and 4PyTP (in erythrocytes) in children with uncomplicated mild to severe chronic renal failure. A preliminary report of some of these data has been presented [6]. We have subsequently measured several putative biosynthetic precursors in plasma and erythrocytes, and it is now clear that 2PY and 4PyTP are derived from NAD^+^ by distinct degradation pathways. Both are biomarkers of the oxidative stress that accompanies renal failure, and their accumulation is a result of poor filtration by the failing kidney. More importantly, metabolites of 4Py-riboside within other tissues may inhibit several enzymes that are crucial for cell division and proliferation. 

## 2. Materials and Methods

### 2.1. Patients with Renal Failure

Children aged 8–17 attending regular out-patient renal clinics or the haemodialysis unit of Great Ormond Street Hospital for Children (GOSH) were invited to provide a small blood sample, after they or their parents had given informed consent. Approval for the study was given by the GOSH Research Ethics Committee. The children (18 girls, 16 boys) had mild to severe chronic renal failure, with no other known metabolic disorder. Recruitment of a further 5 children undergoing peritoneal dialysis was subsequently permitted by the same Research Ethics Committee.

Glomerular filtration rate (GFR, mL/min/1.73 m^2^) in out-patients not receiving artificial dialysis was calculated from the creatinine concentrations in samples of plasma taken on the same occasion and assayed by the Chemical Pathology laboratories at Great Ormond Street Hospital. The National Kidney Foundation paediatric GFR calculator [7] was used: this takes account of the height, age and sex of the child to give a calculated value that is very close to the actual value [8]. The reference value for normal function is a GFR above 75 mL/min/1.73 m^2^ [8]. Of the 34 patients, twelve children were in end-stage renal failure, defined as GFR < 10 mL/min/1.73 m^2^: six patients were attending the Haemodialysis Unit three times per week (HD), and six were receiving continuous ambulatory peritoneal dialysis (PD).

### 2.2. Processing of Blood Samples for Liquid Chromatography

Aliquots of 1 mL heparinised blood were centrifuged at 15,800 g in a microcentrifuge within two hours of venepuncture: the plasma was removed and retained, and the top one-fifth of the cells, containing lymphocytes and platelets, was discarded. Washing of cells and extraction of erythrocytes and plasma by trichloroacetic acid were performed as described previously [3]. Water used in all solutions was freshly double-distilled, and buffers were de-gassed before use, ensuring a very low base-line with a modest rise of absorbance with increasing pH. The extracts were analysed by liquid chromatography with UV diode array analysis (230–310 nm) as described previously [3]: typically we used anion-exchange HPLC for erythrocyte extracts and reversed-phase HPLC for plasma extracts. A mixture of 17 authentic standards was run before each batch of samples, and conditions (gradient or elution rate) were adjusted to ensure separation of all the nucleotides and nucleosides of interest. 

Concentrations (µM) of the compounds in the cytoplasm of the erythrocytes were calculated using the known response factor in the HPLC (calculated from standard solutions; for 4PyTP the molar extinction coefficient at 254 nm was calculated from solutions of known extinction and dry weight), the volume-equivalent of packed erythrocytes in the extracts applied to the column, and an estimate that 67% of the packed cell volume corresponds to cytoplasm. Our data therefore relate to the content of mature erythrocytes, and not to the actual haematocrit of the original blood samples.

### 2.3. Identification of the Pyridine and Pyridone Bases and 4Py-Ribonucleoside in HPLC Profiles

**Figure 1:**
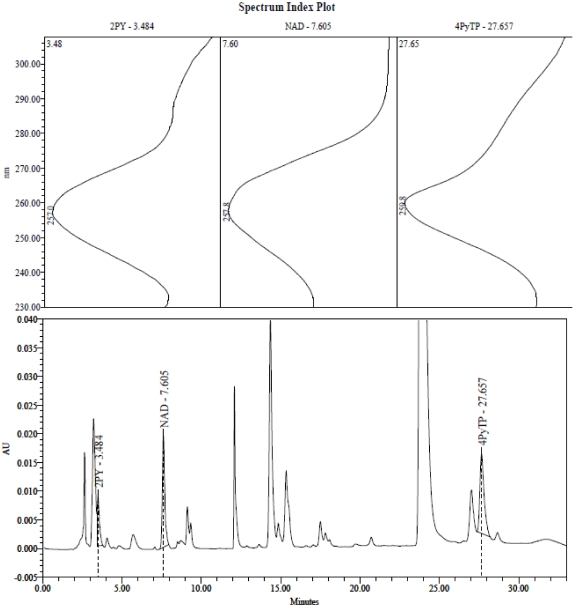
Anion exchange HPLC analysis of erythrocyte extract from a patient in the “peritoneal” group. Nucleotides were separated on a Waters trimodule system with PDA detection from 230–310 nm. Extract (12 µL) was injected on to a 5 µm NH2-2 hypersil column (250 × 3.2 mm) ( Phenomenex) with a linear gradient from 100% Buffer A (5mM sodium phosphate, pH 3.0), to 40% Buffer B (0.5 M potassium phosphate, 1.0M potassium chloride, pH 3.3) over 25 min, a run time to 33 min, and a flow rate of 0.5 mL/min. Peaks corresponding to 2PY (3.48 min), NAD (7.60 min) and 4PyTP (27.65 min) are labelled, and their UV spectra are shown in the upper panels. ATP is the large peak eluting at approx 23.8 min: GTP elutes at 26.9 min.

The elution profile of nucleotides analysed in anion-exchange HPLC has been well-characterised. The nucleotide that has now been identified as 4PyTP elutes after the distinctive peaks of ATP and GTP at the end of the rising gradient (see Figure 1). 

**Figure 2:**
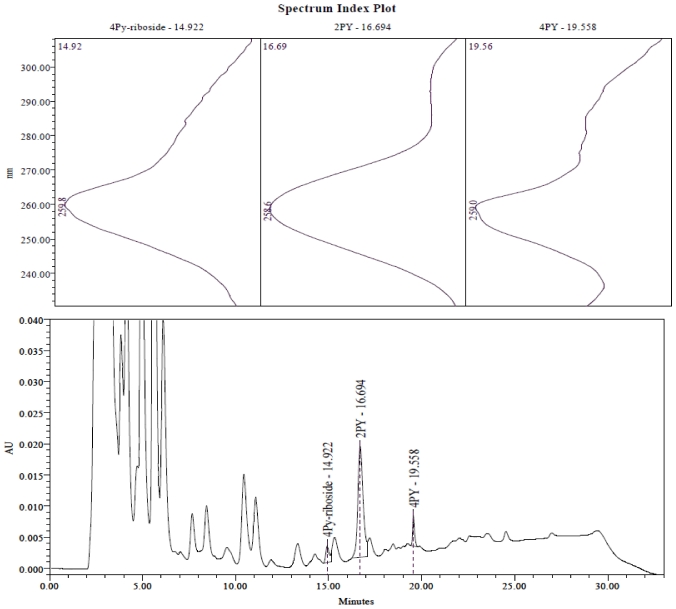
Reverse-phase HPLC analysis of plasma from a patient in the “peritoneal” group. The nucleosides and bases were separated by reverse phase HPLC, on a Waters Alliance system with PDA detection from 230–310 nm. Extract (25 µL) was injected on to a 5 µm ODS-1 Hypersil column (250 × 3.2 mm) from Hichrom Ltd, with a linear gradient from 100% Buffer A (40 mM ammonium acetate pH 5.0), to 20% Buffer B (methanol:tetrahydrofuran:acetonitrile (80:10:10)) in 25 min, with a flow rate of 0.5 mL/min and a run time of 33 min. Peaks corresponding to 4Py-riboside (14.92 min), 2PY (16.69 min) and 4PY (19.56 min) are labelled on the chromatograph, and their UV spectra are shown in the upper panels.

The methylated pyridone bases were identified previously [3,4] by comparison with the NMR and mass spectrometric analysis of chemically synthesized 2PY and 4PY, and recognized by their characteristic UV absorption spectra (230–310 nm) and retention times in reversed-phase HPLC (see Figure 2). 

The ribonucleoside of 4Py eluted at 14.5–15.5 min from the reversed-phase HPLC system used for plasma, and at 3.2–3.5 min when applied to the anion-exchange HPLC system used for analysis of the nucleotides. Its identity was confirmed by LC-MS as described below. The absorption spectrum of 4Py-riboside was identical with a synthetic version, shown in supplementary material to reference 5; the nucleotides have similar spectra [3,5]. 

### 2.4. Analysis of Plasma Samples by LC-MS

A novel assay [5], using reverse-phase HPLC and mass detection, enabled closer examination of 4Py-riboside, nicotinamide and *N*^1^-methyl nicotinamide in plasma samples. The identities suggested from the characteristic UV spectra or retention times were confirmed by fragmentation pattern and mass detected in the positive ion mode.

## 3. Results and Discussion

### 3.1. Degree of Renal Failure and Uremia

The children in this study all had poor kidney function, shown by decreased filtration and the accumulation of creatinine, urea and uric acid in the plasma, which may affect the liver, kidney, brain and other organs. Similarly, metabolites of NAD^+^ breakdown accumulated in parallel with the degree of renal failure because of poor filtration by the kidney (Table 1). In the children with the lowest degree of renal failure (calculated GFR above 72 mL/min/1.73 m^2^), urea concentrations fell within the normal reference range but creatinine values were elevated.

Children in end-stage renal failure, defined as GFR < 10 mL/min/1.73 m^2^, received haemodialysis (HD) three times per week, or continuous ambulatory peritoneal dialysis (PD). Our blood samples were taken before haemodialysis, usually 2.5–3 days since the previous session; in this group (*n* = 6, “haemodialysis”) the plasma concentrations of urea and creatinine were 5 to 10 times the levels found in healthy children. The PD patients (*n* = 6, “peritoneal”) were attending the morning out-patient clinic and thus were between bag changes; urea concentrations resembled those in children with moderate-severe renal failure, but creatinine concentrations were grossly elevated (Table 1).

Uric acid concentrations in our patients were universally elevated above the reference ranges, especially in the plasma of HD patients (Table 1), but there was no correlation with GFR in the non-dialysed patients (*r* = −0.077) and no significant difference between values in dialysed and non-dialysed patients (Mann-Whitney test).

### 3.2. Correlation of Methylated Pyridones and Tryptophan in Plasma with Severity of Renal Failure

In the non-dialysis patients, there was a close correlation between the GFR and the logarithm of 2PY concentration (*r* = −0.783, significant at the 0.01 level), and 4PY also accumulated but at much lower concentrations. 

Dialysis patients had creatinine concentrations above 600 μM, and 2PY above 30 μM, at the time of study. These values did not overlap with those of the other patient groups, who were well controlled with creatinine values below 400 μM, and 2PY below 15 μM. The difference between the 2PY concentrations in the plasma of the dialysis patients and the non-dialysed patients was significant (*p* < 0.0005, Mann-Whitney test). Compared with children with low-normal renal function the concentrations of 2PY and 4PY were increased 13-fold and 39-fold respectively for the PD group, and 20-fold and 58-fold for the HD group (Table 1).

Circulating levels of tryptophan, the amino acid precursor for *de novo* NAD^+^ biosynthesis, correlated slightly with GFR (*r* = 0.574), being highest in the plasma of children with a high GFR and undetectable in some of the haemodialysis patients. Circulating tryptophan concentrations are known to fall in haemodialysis patients [9,10,11], and chronic renal failure causes a depletion of tryptophan in the plasma [12]. It has been reported that metabolites such as L-kynurenine and quinolinate from the *de novo* tryptophan-NAD^+^ biosynthetic pathway [13,14] accumulate as a result of oxidative changes in the liver and kidney [15,16], displace the binding of tryptophan to human serum albumin [17,18] and contribute to some of the symptoms of uraemia in the circulation and brain [10,11,19,20]. The measurement of these metabolites was outside the scope of this work. Total tryptophan values (free and protein-bound) in the plasma of healthy adults are reported to be 31.2 ± 7.4 μM [20], which was matched only by the children with lowest renal failure (Table 1).

**Table 1:** Contents of plasma from children with renal failure.

	Low-Normal *n* = 5	Mild *n* = 9	Mod-Severe *n* = 8	Peritoneal *n* = 6	Haemodialysis *n* = 6
GFR	95.00 ± 20.6	59.22 ± 5.26	34.75 ± 12.83	≤10	≤10
Creatinine	95.8 ± 27.8	140.1 ± 27.4	248.5 ± 116.9	839.2 ± 223.9	1005.3 ± 258.6
Urea	4.66 ± 1.25	8.42 ± 3.33	14.85 ± 5.19	13.80 ± 2.83	23.22 ± 3.03
Uric acid	374.4 ± 56.4	454.3 ± 26.6	439.1 ± 19.5	426.2 ± 29.4	545.2 ± 51.1
Tryptophan	31.79 ± 6.58	29.41 ± 8.21	18.80 ± 8.89	10.53 ± 6.84	4.96 ± 7.75
2PY	2.55 ± 1.05	6.84 ± 3.38	10.84 ± 3.32	34.90 ± 8.23	53.38 ± 23.60
4PY	0.16 ± 0.23	1.06 ± 0.79	1.77 ± 0.94	6.45 ± 1.01	9.50 ± 3.06
Nicotinamide	0.38 ± 0.30	0.44 ± 0.19	0.57 ± 0.29	0.58 ± 0.40	0.94 ± 0.77
N-Me-nic	0.05 ± 0.03	0.08 ± 0.03	0.06 ± 0.03	0.06 ± 0.03	0.14 ± 0.07
4Py-riboside	0.03 ± 0.04	0.08 ± 0.08	0.10 ± 0.12	0.42 ± 0.23	0.28 ± 0.26

Patients with chronic renal failure were assigned to groups according to calculated GFR or dialysis mode. The non-dialysis patients were divided empirically into three approximately equal groups: “low-normal renal function” with GFR ≥ 71 mL/min/1.73 m^2^ (actual range 72–120 mL/min/1.73 m^2^); “mild renal failure” with GFR 51–70 mL/min/1.73 m^2^ (actual range 53–69 mL/min/1.73 m^2^) and “moderate-severe renal failure” with GFR ≤ 50 mL/min/1.73 m^2^ (actual range 17–50 mL/min/1.73 m^2^). The reference ranges for compounds in the plasma are: 2.5–6.0 mM urea for children aged 1–13 (2.5–7.5 mM over 13 years); 30–65 μM creatinine for children aged 6–9 years, and 35–90 (100) μM for girls (boys) of 14–18 years. The reference range for uric acid in children of 7–10 years is 120–295 μM; at 12–16 years 180–345 μM (girls), 160–465 μM (boys). Each group (see Table 1 and Table 2) contains children across the entire age range; The normal range of 2PY concentrations has been calculated, using a slightly different detection system, as 0.39 ± 0.22 µM in plasma of healthy children aged 5–16, and 1.01 ± 0.5 µM in adults [10] while in our previous study [3] we estimated 9.0 ± 4.5 µM in the plasma of healthy adults. Metabolites (mean ± SD, concentrations expressed in μM, apart from urea mM) were identified and measured by HPLC as described in the text. 2PY and 4PY, *N*^1^-methylated 2-pyridone and 4-pyridone respectively; N-Me-nic, *N*^1^-methylnicotinamide.

Nevertheless, the overall levels of NAD and NADP in the erythrocytes (Table 2) were not altered in the way expected if circulating tryptophan were a significant source for *de novo* synthesis. It was not possible to measure tryptophan stores in muscle or in the liver, where much of the NAD^+^ synthesis takes place: however the children were well-nourished and would obtain both tryptophan and NAD^+^ precursor vitamins [21] from the diet for *de novo* and salvage synthesis respectively. 

#### 3.2.1. Renal Failure and the Accumulation of Methylated Pyridone and Pyridine Bases

It is known that excess nicotinamide from the diet or released from NAD^+^ (e.g., through the activity of PARP-1, sirtuin or NAD^+^ glycohydrolase), if not used directly in recycling, is detoxified by methylation at the *N*^1^ position [22]. 

**Scheme 1:**
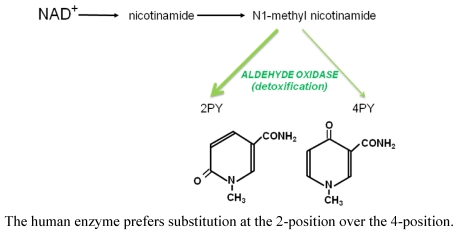
Detoxification of excess nicotinamide.

Subsequent attack by aldehyde oxidase in the human liver (Scheme 1) modifies *N*^1^-methylnicotinamide preferentially at the 2 position of the pyridine ring, resulting in a much higher concentration of 2PY than 4PY in the plasma and urine [23,24] as seen in Table 1. It can be seen that haemodialysis patients, who have the highest 2PY and 4PY values, also carry high concentrations of nicotinamide and *N*^1^-methyl nicotinamide in the plasma, suggesting that the capacity of the oxidase reaction has been saturated.

Aldehyde oxidase is known to have a wide range of potential substrates, and the site of oxidation of nicotinamide metabolites, for example, is moderated by substitution(s) elsewhere in the pyridine ring [25]. Concentrations of *N*^1^-methylnicotinamide and the methylated pyridones, 2PY and 4PY, are usually low but detectable in the plasma of healthy subjects; they circulate freely in plasma without being bound to a carrier protein such as albumin. The observed rise in concentrations according to age reflects the known decline in renal function in adults [26], since these soluble metabolites would usually be excreted in the urine [27].

#### 3.2.2. Presence of 2PY in Erythrocytes

A peak eluting at 3.3 to 3.5 min from our anion exchange HPLC system has now been identified as 2PY (it was erroneously reported earlier [6] to be the ribonucleoside related to 4PyTP) and has been found in the erythrocytes of all dialysis patients (Figure 1), but in only a small number of erythrocyte extracts from non-dialysed patients. In dialysis patients, where the concentration of 2PY in the plasma was already above 30 μM (Table 2), the concentration of 2PY in the red cells of each patient correlated closely with the external concentration in the plasma (*r* = 0.845). It should be noted that no 2Py-nucleoside has been observed.

### 3.3. Concentration of Nucleotides in Erythrocytes: Correlation with Severity of Renal Failure

As noted previously, 4PyTP remained in erythrocytes after artificial dialysis [3] and in this study we also confirm that high concentrations of 4PyTP correlate with decreases in the calculated GFR in children and are highest in dialysis patients (*p* < 0.0005 between dialysis and non-dialysis patients). As in our previous report on adult patients, the children undergoing peritoneal dialysis had very high mean concentrations of 4PyTP (200 μM) and 4PyDP (10 μM). Values for 4PyTP in the HD and PD groups differed significantly (*p* < 0.002) in the Mann-Whitney non-parametric test, being 3.7 and 9.7 times the concentration for children with low-normal renal function (Table 2).

Table 2 shows that concentrations of other nucleotides were also elevated in erythrocytes: ATP showed a small rise associated with degree of renal failure, with a greater increase in the PD group. Concentrations of GTP were elevated in both dialysis groups. 

**Table 2:** Nucleotides in the erythrocytes of children with renal failure.

	Low-Normal *n* = 5	Mild *n* = 9	Mod-Severe *n* = 8	Peritoneal *n* = 6	Haemodialysis *n* = 6
4PyTP	20.76 ± 5.85	28.96 ± 13.49	55.70 ± 31.28	200.57 ± 85.11	76.62 ± 25.00
4PyDP	1.32 ± 1.44	2.34 ± 1.73	7.26 ± 6.59	10.38 ± 6.00	4.48 ± 5.42
2PY	n.d.	n.d.	n.d.	10.5 ± 4.36	11.72 ± 8.46
NAD	56.82 ± 6.79	59.74 ± 7.05	67.81 ± 6.16	72.90 ± 9.81	76.48 ± 13.35
NADP	43.54 ± 2.61	48.8 ± 6.61	46.24 ± 3.53	47.55 ± 10.93	45.12 ± 4.40
GTP	44.78 ± 2.62	44.31 ± 3.23	45.26 ± 4.82	88.63 ± 12.74	81.78 ± 27.57 *
ATP	1222 ± 172	1350 ± 261	1486 ± 197	1754 ± 306	1449 ± 310 *

* Omitting values from one patient, who was ITPase-deficient; Patients with chronic renal failure were assigned to groups according to calculated GFR or dialysis mode. See Table 1 for details; Metabolites (mean ± SD, concentrations all expressed in μM) were identified and measured by HPLC as described in the text. 2PY, *N*^1^-methylated 2-pyridone; 4PyTP and 4PyDP, triphosphate and diphosphate of 4-pyridone-3-carboxamide-1β-D-ribonucleoside; n.d., not detected; NAD and NADP refer to sum of oxidized and reduced forms; The concentrations of nucleoside triphosphates in erythrocytes of healthy adult volunteers and unaffected relatives of renal patients [3] were 8.1 ± 3.4 µM 4PyTP, 39.0 ± 10.9 μM GTP and 1229 ± 184 μM ATP.

### 3.4. Measurement of 4Py-Riboside in Plasma

Plasma samples were analysed for 4Py-riboside using the LC-MS system [5], which enabled us to confirm the identity of the compound observed in the RPLC separation (Figure 2). There was a small increasing trend related to the degree of renal failure in the non-dialysis patients, and the compound was found at distinctly higher concentrations in the plasma of dialysis patients (Table 1). Children in the PD group had higher concentrations of the riboside in their plasma (0.42 ± 0.23 μM) than in the HD group (0.28 ± 0.26 μM), compared with 0.03 ± 0.04 μM for non-dialysed patients with severe renal failure.

#### 3.4.1. Correlation of 4Py-Riboside and 4PyTP Concentrations

During the analysis of 4PyTP [5], pooled and purified from repeated HPLC runs of erythrocyte extracts of an adult PD patient, the ribonucleoside (4Py-riboside) was the sole digestion product after treatment with snake venom 5'-nucleotidase with or without purine nucleoside phosphorylase [28]. This indicates that the 4Py-riboside (unlike NR [29,30]) is not degraded to the base by the phosphorylase, and explains why the related base, 4-pyridone-3-carboxamide, was never seen in the circulation. We have established that 4Py-riboside is the authentic precursor of 4PyTP [5], being converted to the nucleotide after uptake into human erythrocytes. Thus the ribonucleoside circulates in the plasma until taken up by the erythrocytes, and its concentration is reflected in the higher concentration of 4PyTP in dialysis patients. 

4Py-riboside, described as PCNR (4-pyridone carboxamide ribonucleoside), has been isolated elsewhere from urine of leukaemia patients [31]. It occurs in the serum of reference populations in Italy and the USA [32] at approximately 60 nM, similar to the non-dialysed children in our study (Table 1). Its presence in the urine of patients with healthy kidneys is considered to be derived from turnover of NAD^+^ and NADP, not from dietary nicotinamide [33]. In another study, we calculated the excretion of authentic 4Py-riboside as 26.7 ± 18.2 µmoles per day, equivalent to 13.6 ± 9.3 mg per day, from nine healthy subjects [5]. Elevated concentrations of 4Py-riboside, along with breakdown products of tRNA, report on the increased turnover of cells associated with cancer [34]. Patients with advanced HIV infection also have elevated plasma concentrations of approximately 74 nM [35], but this is much lower than the mean values of 420 nM in our PD patients and 280 nM in the HD group (Table 2). The higher concentration in the plasma of the former, compared with higher 2PY concentrations in HD patients, suggests that these two compounds are filtered differently, and also that 4Py-riboside is protein-bound in the plasma. In patients relying upon artificial dialysis, we observe that all intermediates accumulate to such high concentrations that 2PY and 4Py-riboside enter the erythrocytes, but 2PY is not converted further. The ribonucleoside is taken up rapidly and phosphorylated within the erythrocytes: the highest concentration of 4PyTP is found in the erythrocytes of PD patients, who also have the greatest relative increase of circulating 4Py-riboside. 

Normal breakdown of erythrocytes will release NAD^+^ catabolites such as 4Py-riboside [34,35] and 4PyTP, which may be converted back to 4Py-riboside in the plasma [5]; however, in the absence of normal excretion via urine, the intact erythrocytes in dialysis patients may pick up the circulating ribonucleoside and convert it back to 4PyTP. This model could explain why 4PyTP increases less in HD erythrocytes (3.7-fold) compared with the 4Py-riboside ratio (9.3-fold) in plasma, since mechanical haemodialysis is known to damage red cells, which thus also have a younger profile than the erythrocytes in PD patients. 

#### 3.4.2. Origins of 4Py-Riboside

We have observed, through analysis of many clinical samples over 25 years (LDF, HAS), that both 2PY and 4PyTP are absent from the blood of subjects deficient in molybdenum cofactor, which is essential to the oxidases [36,25]; this strongly implicates aldehyde oxidase activity in the conversion of both methylated and ribosylated pyridines into the respective pyridones. A representative clinical sample from a MoCo-deficient infant is illustrated in reference 3. In humans *N*^1^-methylnicotinamide is converted to 2PY in great excess over 4PY [6,25], whereas the reverse is true in other mammals. Even when 4Py-riboside is found in significant concentrations in the plasma of renal failure patients, especially those with high levels of 4PyTP, we have not detected a 2-pyridone analogue. Early reports of the 2Py-riboside [22] were not confirmed, and a chemical analysis was not given; it may instead have been 4Py-riboside that was excreted at the rate of 5–10 mg per day in the urine of healthy adults, as in our study [5]. 

**Scheme 2:**
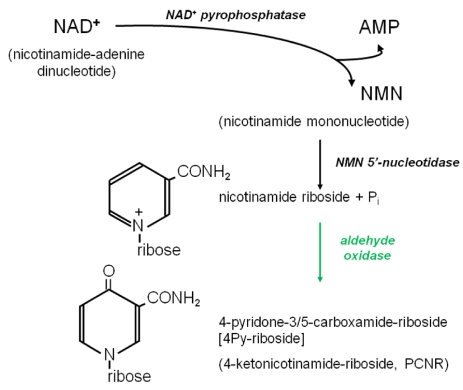
Role of aldehyde oxidase in the generation of 4Py-riboside.

Nicotinamide riboside (NR) is a chemically similar compound; it arises in a parallel pathway for NAD^+^ breakdown in which NMN is released by the action of NAD^+^ pyrophosphatase (EC 3.6.1.22) on NAD^+^. This enzyme, and the NMN phosphohydrolase (EC 3.1.3.5) which generates the nicotinamide riboside, have been reported in human serum and may originate from the liver [37,38]. Alternatively, nicotinamide riboside (NR) has been described as a novel nutrient in milk [21], and may be excreted in the bile, if in excess [39]. It is a substrate for several enzymes in pathways leading to NAD^+^ formation [21] and is thus a precursor vitamin. We have proposed a pathway (Scheme 2) in which NR is oxidised at the 4-position by aldehyde oxidase to yield 4Py-riboside. In healthy humans, the oxidation could also be a detoxification step, avoiding accumulation of excess nicotinamide, as in Scheme 1.

We have no direct evidence that aldehyde oxidase can convert NR into a pyridone riboside, nor that the 4-position is the only site of attack. However, in studies of the DNA methylation inhibitor zebularine (a pyrimidinone ribonucleoside), aldehyde oxidase from several mammalian species is shown to act at the 4-position of the pyrimidine ring, producing uridine [40]; this reaction is a direct parallel of the oxidation reaction proposed in Scheme II. It is entirely plausible that the presence of the ribose at N-1 protects C-2 in NR from attack by human aldehyde oxidase. An alternative scheme is conceivable: oxidation of NAD^+^ followed by direct cleavage by pyrophosphatase to generate 4Py-riboside.

### 3.5. The “Paradoxical” Elevation of GTP Concentrations

In this study, we observed elevated concentrations of GTP and ATP in the erythrocytes of dialysis patients (Table 2), where 4PyTP concentrations were highest. This is reminiscent of our study of adult renal transplant patients after several months’ immunosuppressive therapy with mycophenolate mofetil, where we observed “paradoxical” high levels of GTP (approx 146 μM) in the erythrocytes [41]. We consider this to be a paradox since in other cells in the presence of mycophenolate mofetil, such as mononuclear leucocytes [42], the GTP concentrations are depleted: this is the expected result of the drug’s role as an inhibitor of the enzyme inosine monophosphate dehydrogenase (IMPDH), which is crucial in the synthesis of GTP and ATP. A similar increase in GTP (attributed to “induction” of IMPDH) was observed in patients treated with ribavirin [43]. We proposed [41] that structural stabilisation, as described by Nimmersgen *et al*. [44], of the IMPDH-inhibitor complex allowed a continuation of enzyme activity in non-nucleated mature erythrocytes, where new enzyme could not be synthesised. The analogy in this study would be that 4Py-riboside or the 4Py-nucleotides in patients with renal failure may bind specifically to IMPDH, stabilising the structure and activity in maturing erythrocytes, thus allowing GTP levels to increase slowly in these cells. 

We were able to perform only a partial enzymological assessment of the interaction of the 4Py-riboside and 4Py-nucleotides with IMPDH (purified from platelets; EAC, LDF preliminary results) because the putative inhibitors were in limited supply. We would predict that renal failure patients with high 4PyTP concentrations in the erythrocytes would have a higher IMPDH activity in these cells, alongside depleted GTP and IMPDH in lymphocytes. *In vitro*, one could study the interaction of the riboside and nucleotides with IMPDH using the biophysical methods described by Nimmersgen *et al*. [44].

### 3.6. Further Metabolism of 4Py-Ribonucleoside

It has previously been demonstrated that 4Py-riboside is taken up into intact human erythrocytes *in vitro* in a reaction carried out by adenosine kinase since it is inhibited by 5'-iodotubericidin, with a net consumption of ATP, and is rapidly converted to the mononucleotide and 4PyTP [45]. The erythrocyte samples in this study showed a very high accumulation of 4PyTP and 4PyDP, reflecting the much longer timescale and equilibrium within cells obtained *in vivo*. Further studies in rodents have come to the intriguing conclusion that 4PyTP may be found only in erythrocytes: only 4PyMN was detected in the numerous other tissues that were investigated [46].

Once again we are drawn to a comparison of 4Py-ribonucleotides and the known inhibitors of IMPDH. These inhibitors are especially useful in immunosuppression and the treatment of leukaemia because this enzyme regulates cell proliferation through the supply of the purine nucleotide GTP. Pharmaceutically-active ribonucleoside analogues are pro-drugs, falling broadly into two classes, according to whether they are converted *in vivo* principally into toxic analogues of NAD^+^ (tiazofurin, benzamide riboside) or into nucleoside mono-, di- and triphosphates (ribavirin, viramidine) which compete at the IMP binding site [47,48,49,50,51]. Sensitivity to these analogues therefore depends upon the expression in the cells of IMPDH [52], and requires NMN adenyltransferase [37,51,53] for conversion of the ribonucleoside to the dinucleotide. In sensitive lymphocytes, the concentration of GTP falls to unsustainably low levels [51,52] in the presence of tiazofurin anabolites.

Tiazofurin is taken up into cells by facilitated nucleoside transport [53,54], competing with uridine. Further metabolism to the adenine dinucleotide TAD (the toxic NAD^+^ analogue) occurs in lymphocytes but, crucially, not in erythrocytes [55]. The NAD^+^ analogue is a more effective inhibitor of IMPDH at low concentrations (*K_i_* 0.13 µM). The ribonucleoside is phosphorylated by one or more of three enzymes [47]: adenosine kinase, NR-kinase or cytosolic 5'-nucleotidase. This is very similar to the conclusions we have drawn (above) about the further metabolism of 4Py-riboside. Conversion of tiazofurin to TAD [55,56] was observed in different cell types which were outside the scope of this study, and would therefore prevent us from observing a similar conversion of 4Py-riboside to an NAD^+^ analogue. Within erythrocytes, nucleotides of ribavirin [49] and tiazofurin [57] have been shown to contribute to haemolytic anaemia, which is also a feature of uraemia.

**Scheme 3:**
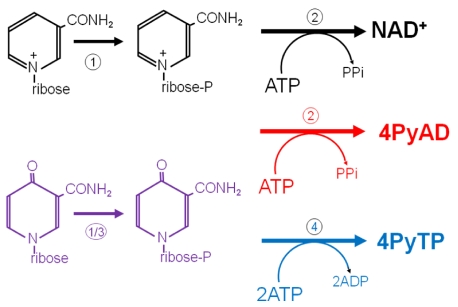
Further metabolism of 4Py-riboside to 4PyTP and to an NAD^+^ analogue.

In Scheme 3 we summarise our current thinking about the further metabolism of 4Py-riboside, comparing it with the recycling of nicotinamide riboside (NR) and its conversion into NAD^+^ (shown in black on the top line). We propose that 4Py-riboside enters erythrocytes and other cells and is immediately phosphorylated (shown in purple) by NR kinase (1) or adenosine kinase (3) to provide the 4PyMN for further steps. Since NMN is converted to NAD^+^ by (2) NMN-adenyltransferase [37], we are proposing that 4Py-riboside may also be converted to the NAD^+^ analogue, here named as 4PyAD, by a similar reaction (shown in red) in lymphocytes and other cells. We are confident that in erythrocytes the mononucleotide is phosphorylated (4) to the nucleoside triphosphate, which accumulates to extremely high concentrations in end-stage chronic renal failure.

## 4. Conclusions

The compound that we have conclusively identified as 4-pyridone 3/5-carboxamide ribonucleoside triphosphate (4PyTP) was originally designated a “uremic toxin” on the basis of its notable accumulation in a series of blood samples obtained from patients with chronic renal failure. The accumulation in the erythrocytes set it apart from the well-known methylated pyridones 2PY and 4PY, which are prominent in the plasma of renal failure patients. Like 2PY and 4PY, however, it is the product of the oxidizing conditions arising in renal failure, and its absence from erythrocytes of MoCo-deficient patients supports the mechanism involving aldehyde oxidase action on the nicotinamide riboside. This mechanism could be tested (if ethically approved) by treating patients with raloxifene, or 5-benzylacyclouridine, both of which prevented the action of aldehyde oxidase on zebularine [40].

The toxic nature of the nucleotides is suggested by our evidence for specific binding of 4Py-riboside and 4PyMN to IMPDH from human platelets, leading to a small but consistent activation at the concentrations found in the erythrocytes of dialysis patients (4PyTP was not tested in this assay). Our work continues to elucidate whether, like tiazofurin, a more powerful toxicity is caused by an analogue of NAD^+^ in cells of the immune system [55], or by hemolytic effects of the nucleoside triphosphate in the erythrocytes [57]. 

## Drugs Implicated in Inhibition of IMPDH

Benzamide riboside, 1-β-D-ribofuranosylbenzene-3-carboxamide; MMF, mycophenolate mofetil, the morpholinoethyl ester prodrug of mycophenolic acid; Ribavirin, 1-β-D-ribofuranosyl-1,2,4-triazole-3-carboxamide; TAD, thiazole-4-carboxamide adenine dinucleotide (*cf* NAD); Tiazofurin, 2-β-D-ribofuranosylthiazole-4-carboxamide; Viramidine, 1-β-D-ribofuranosyl-1,2,4-triazole-3-carboxoamidine.
